# Common femoral artery transection during total hip arthroplasty for a neglected septic hip dislocation: a case report

**DOI:** 10.1097/RC9.0000000000000847

**Published:** 2026-08-20

**Authors:** Zied Masmoudi, Sami Bahroun, Alaa Aloui, Ameur Triki, Karim Mallek, Mohamed S. Daghfous

**Affiliations:** Adults B Department, Kassab Institute of Orthopedics, Mannouba, Tunisia

**Keywords:** acute limb ischemia, case report, total hip arthroplasty, vascular injury

## Abstract

**Introduction:**

Vascular injury during total hip arthroplasty (THA) is a rare but life-threatening complication. Severe anatomical distortion, particularly in complex hip pathology, may substantially increase this risk.

**Case presentation:**

A 62-year-old woman with a history of childhood septic arthritis presented with a chronically dislocated hip and advanced joint destruction. During THA, a sudden massive hemorrhage occurred during acetabular exposure. The postoperative absence of distal pulses and a sensorimotor deficit raised suspicion of acute limb ischemia. Computed tomography (CT) angiography confirmed occlusion of the femoral artery. Emergency vascular exploration revealed complete transection of the common and superficial femoral arteries, which were reconstructed using a polytetrafluoroethylene graft.

**Clinical discussion:**

Neglected septic hip dislocation profoundly alters local anatomy, bringing major vessels into close proximity to the acetabulum and increasing the risk of vascular injury. Early recognition of abnormal bleeding and postoperative vascular compromise is essential. Close collaboration between orthopedic, anesthesiology, and vascular teams is critical to achieving limb salvage and preventing fatal outcomes. Selective use of CT angiography can aid preoperative risk assessment in hips with severe anatomical distortions.

**Conclusion:**

Vascular injury during THA is a rare but serious complication. Meticulous surgical technique, systematic postoperative vascular examination, and prompt multidisciplinary intervention are critical to optimize survival and limb salvage.

## Introduction

Arterial injury during total hip arthroplasty (THA) is rare but life-threatening. This study presents a case of a femoral artery section during THA; we describe the intra-operative management, the steps for diagnosis, and the multi-disciplinary approach to this complication. We also suggest tools for prevention and risk assessment. This work has been reported in line with the SCARE criteria[1].

## Case presentation

We present a case of a middle-aged woman with a history of septic arthritis of the left hip at age 2 that was neglected and treated with antibiotics until a fistula formed, which eventually healed after 1 year.

The patient had had a limp and intermittent pain since childhood, but no fever or purulent drainage. Her symptoms were managed with pain medication.

HIGHLIGHTS

- Total hip arthroplasty is a commonly performed procedure in orthopedic surgery.
- Femoral artery injury during total hip arthroplasty is exceptionally rare but catastrophic.
- A neglected septic hip dislocation may profoundly distort the local anatomy and vascular relationships.
- Computed tomography angiography can aid risk assessment and surgical planning.
- Early recognition and multidisciplinary management are critical to survival and limb salvage.

She presented to our hospital at the age of 62 with persistent pain, a limp, and a limited range of motion of the left hip. On physical exam, she had no fever, and the fistula was dry. Pain was located in the left groin. She had 90° flexion and 20° internal rotation. The leg-length discrepancy was 3 cm.

**Figure 1.**
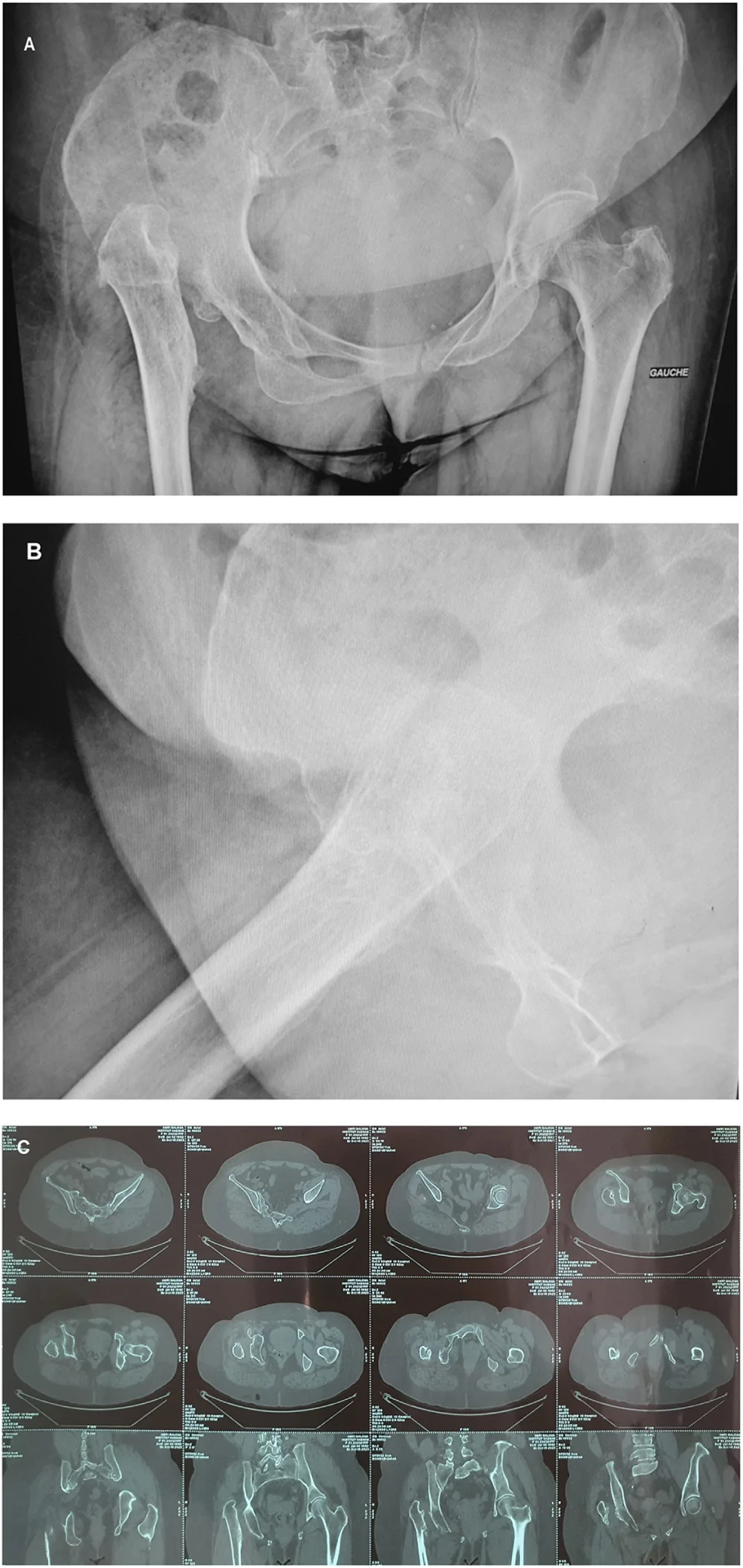
Preoperative anteroposterior (A) and lateral (B) radiographs of the pelvis showing a Crowe IV anterior hip dislocation. CT scan (C) showing complete resorption of the femoral head and neck and a dysplastic acetabulum.

Laboratory results showed no signs of infection. Imaging with plain radiography (Fig. 1) and computed tomography (CT) showed complete resorption of the femoral head and a Crowe IV hip dislocation.

The patient underwent THA via a posterolateral approach; sequential soft-tissue releases were performed to facilitate femoral distalization and reduction. Attention was then directed to the true acetabulum, which was covered in fibrosis. A Hohmann retractor was placed on the anterior column, and careful electrocautery dissection was used to expose the acetabulum.

When tension on the Hohmann retractor was loosened to reposition it, severe bleeding occurred from an area of fibrosis near the true acetabulum. The anesthesiologist was alerted. IV fluid resuscitation, ephedrine, and tranexamic acid were administered. Maintaining tension on the Hohmann retractor significantly reduced the bleeding, which allowed us to dissect and ligate a large vessel. This was necessary to control the bleeding and save the patient’s life, considering we did not have a vascular surgery department at our institution.

After ensuring good hemostasis, we proceeded to ream slightly above the true acetabulum, where a cemented cup was fitted. For the femur, an uncemented dysplastic femoral stem with a short neck was used. The prosthesis was stable, with a good range of motion and a leg-length discrepancy of only 0.5 cm.

Postoperatively, the patient was hemodynamically stable. She had lost an estimated 900 mL of blood but did not require transfusion. However, we noted the absence of a distal pulse and a sensorimotor deficit of the left lower extremity. Acute limb ischemia was suspected.

**Figure 2.**
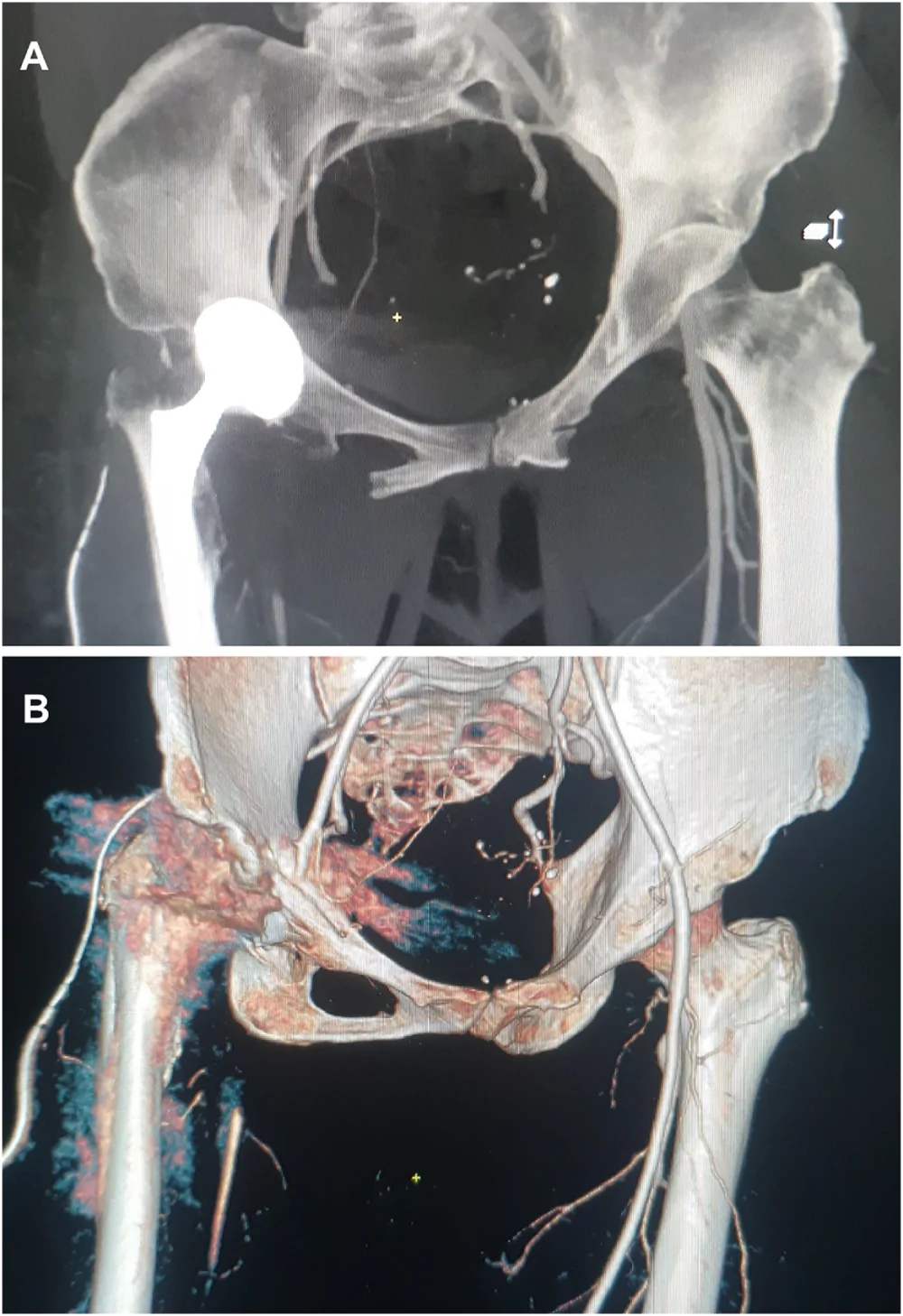
Coronal view (A) and 3D reconstruction (B) from CT angiography showing the absence of opacification in the common femoral artery at the level of the anteroinferior wall of the acetabulum, extending over 100 mm in the proximal third of the superficial femoral artery.

Doppler ultrasound and CT (Fig. 2) confirmed the diagnosis and showed absence of opacification of the common femoral artery at the level of the anteroinferior wall of the acetabulum, extending for over 100 mm up to the proximal third of the superficial femoral artery.

The patient was transferred 4 hours postoperatively to a center with a cardiovascular department, where she was operated on urgently.

Exploration revealed a completely transected common femoral artery that had been tied off at the iliofemoral junction. The superficial femoral artery was transected, severely retracted, and thrombosed.

End-to-side anastomoses were made between the common femoral artery and the superficial femoral artery using a 6-mm polytetrafluoroethylene graft.

Postoperatively, the right limb was warm. Dorsalis pedis and posterior tibial pulses were present and symmetric. However, she continued to have a sciatic nerve palsy, with dorsiflexion of the foot impossible against resistance and paresthesia in the dorsal aspect of the foot.

She was discharged with an anti-equinus splint, pain medication, and lifelong direct oral anticoagulants. She was instructed to remain non-weight-bearing for 6 weeks and to start rehabilitation immediately.

**Figure 3.**
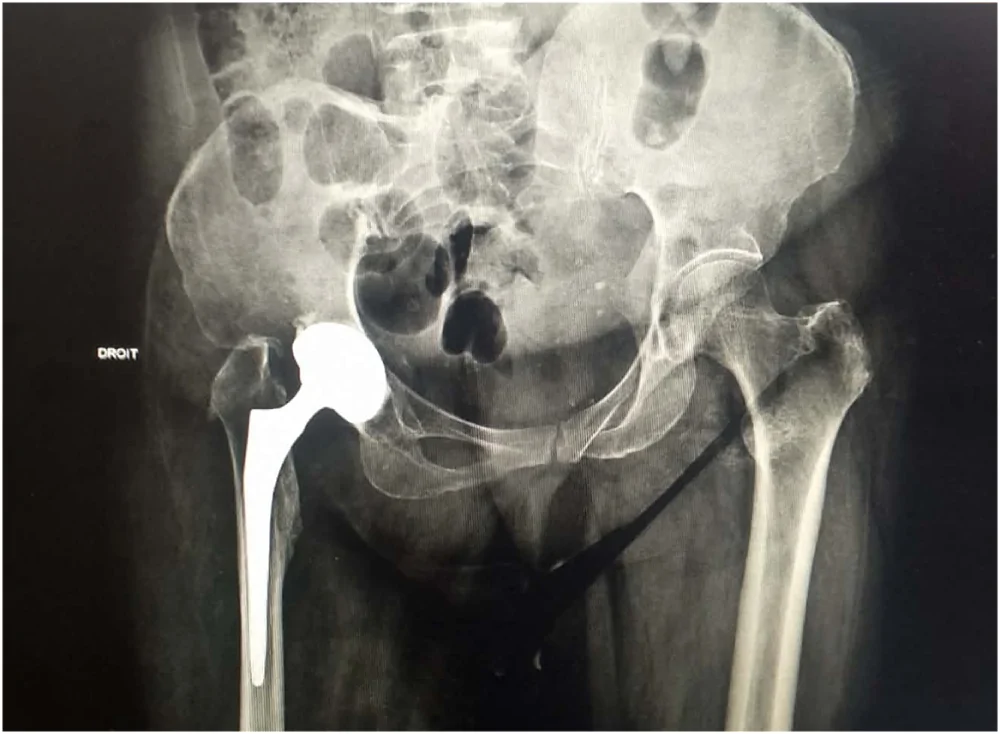
Postoperative anteroposterior pelvic radiograph showing good implant positioning and integration.

At the 3-month follow-up, the patient was walking with one cane, completely pain-free. She had 110° of hip flexion, 40° of abduction, and 30° of internal rotation. The limb had good peripheral perfusion, and the sciatic paresis had resolved. The leg-length discrepancy was minimal and did not require orthotic compensation. The postoperative radiograph showed good implant positioning and integration (Fig. 3).

## Discussion

Vascular injury during THA is very rare, with an incidence ranging from 0.04% to 0.1% in primary THA and 0.2% in revision THA[2].

Reported mechanisms of injury usually include punctures from Hohmann retractors, drills, and acetabular screws, or thermal damage from bone cement or electrocautery[3]. In rare cases, intimal lesions can result from traction, which can be responsible for pseudo-aneurysm formation and delayed perforation[4].

To our knowledge, this is the first case report of a transected common femoral artery. Our patient had a neglected septic hip dislocation with significant anatomical abnormalities, soft-tissue retraction, hip dysplasia, and fibrosis. These are known risk factors for vascular injury in THA[5].

Other risk factors include left-sided THA; Kawasaki analyzed 200 normal hip CT- angiograms and found that vascular structures on the left side were closer to the acetabulum[6].

There is still some controversy about whether routine CT-angiography should be performed before THA. This procedure has been associated with risks of radiation-induced cancers, severe allergic reactions, and contrast-induced nephropathy^[^7,8^]^. However, Diesel recommends preoperative CT-angiography for revision THA to assess the proximity of the vessels to the acetabular component[9].

Complete transection of arteries usually results in their retraction into soft tissues, making visualization and ligation difficult[10]. This was not the case in our patient, probably due to the extensive fibrosis and adhesions. The placement and tension of the Hohmann retractor clamped the vessel proximally and allowed dissection and ligation of the transected end.

Following a massive intraoperative hemorrhage, a major vascular injury should be suspected immediately. Current evidence suggests that once hemostasis is achieved and the patient is hemodynamically stable, prompt vascular assessment with CT angiography is recommended because arterial thrombosis or distal embolization may not be clinically apparent during surgery and can progress to acute limb ischemia[11].

The optimal management strategy remains dependent on the clinical situation. In unstable patients with ongoing hemorrhage, immediate bleeding control takes priority. In contrast, stable patients may benefit from vascular imaging to define the extent of injury and facilitate definitive repair planning[12]. Management should also be individualized according to local resources, availability of an on-site vascular surgeon, and the extent of orthopedic reconstruction already completed. Nevertheless, revascularization should be performed as soon as circumstances allow, as outcomes are highly time-dependent. Restoration of arterial flow within 6 hours is generally considered critical to minimize irreversible soft-tissue and neurological injury[13].

Acute limb ischemia can lead to sciatic palsy, particularly when the ischemic episode is prolonged or severe[14]. Leg lengthening can also be responsible for sciatic palsy[15]. One intraoperative neuromonitoring study reported that nerve lengthening of 5% relative to femoral length was found to be the critical limit[16]. In our case, leg lengthening remained within these limits.

## Conclusion

Vascular injury during THA is a rare but serious complication. A neglected septic hip dislocation can significantly distort local anatomy and alter normal vascular relationships, thereby increasing the risk of vascular injury. Early recognition and multidisciplinary management of this complication are essential for patient survival and limb salvage.

## Data Availability

The data supporting the findings of this study are not publicly available due to patient confidentiality. De-identified data are available from the corresponding author upon reasonable request.
